# Culturally competent care across borders: Implementing culturally responsive teaching for nurses in diverse workforces

**DOI:** 10.1016/j.ijnss.2023.09.001

**Published:** 2023-09-15

**Authors:** Abdulqadir J. Nashwan

**Affiliations:** a Director of Nursing for Education & Practice Development, Hamad Medical Corporation, Doha, Qatar; b Department of Public Health, College of Health Sciences, QU Health, Qatar University, Doha, Qatar

The migration of healthcare professionals, including nurses, is a global phenomenon. It is driven by various factors, including the pursuit of better opportunities, living conditions, and professional development, as well as political instability or conflict in their home countries. The World Health Organization (WHO) has noted that high-income countries often rely on foreign-trained nurses to fill gaps in their healthcare systems [1]. For instance, as of 2021, over 40% (52 million) of all nurses in the United States (US) were expatriates [2]. In the United Kingdom (UK), the percentage of expatriate nurses was even higher, reaching approximately 18% in 2021 [3]. Owing to globalization and migration, healthcare providers must possess cultural competence to deliver improved care [4,5]. Culturally responsive teaching (CRT) is rooted in the idea that culture plays a vital role in shaping people’s behaviors, beliefs, values, and communication styles [6]. Furthermore, these cultural factors influence patients’ perspectives on health, illness, healing, and their preferences for care and communication [7]. By recognizing and embracing these cultural differences, nurses can provide more effective and compassionate care to their diverse patient population [8]. This paper explores the significance of CRT for nurses in diverse multinational workforces and provides examples from various countries, such as the US, Canada, Australia, the UK, Qatar, and Singapore. The previously mentioned countries represent diverse geographical regions and contain multicultural societies. They are representative examples of places where healthcare systems must adapt and cater to a culturally diverse population.

In the US, the Hispanic population is increasing rapidly, emphasizing the need for nursing education in CRT [9]. In addition, Hispanic patients tend to prefer a personal, family-centered approach to healthcare that emphasizes spirituality and traditional healing practices [10]. CRT-trained nurses can acknowledge and incorporate these cultural preferences into their care plans, leading to better patient satisfaction and health outcomes and building trust and rapport between nurses and their patients [11].

Similarly, the Indigenous population in Canada faces significant health disparities and barriers to accessing healthcare services [12]. Culturally responsive nursing care addresses these disparities and builds trust with indigenous patients [13]. For example, nurses working with indigenous populations should know about traditional healing practices and the importance of community and family in the healing process [14]. Nurses can provide more effective and holistic care for their indigenous patients by incorporating these cultural elements into their care plans.

The culturally and linguistically diverse (CALD) population in Australia, including immigrants and refugees, presents unique challenges for healthcare providers [15]. Nurses working with CALD patients must be sensitive to the cultural differences that may impact their patients’ health beliefs, communication styles, and preferences for care [16]. For instance, some CALD patients may hold cultural beliefs about the causes of illness that differ from Western biomedical models or have unique dietary requirements based on their cultural or religious backgrounds [17,18]. By understanding and respecting these cultural differences, nurses can provide more patient-centered care and improve health outcomes for their CALD patients.

The UK, with its diverse population and multicultural society, also highlights the importance of CRT in nursing practice [19]. Nurses in the UK must be prepared to work with patients from various cultural backgrounds, including those from South Asian, African, and Eastern European communities [20]. For example, some South Asian patients may prefer to receive care from healthcare providers of the same gender or have specific dietary restrictions based on their religious beliefs [21]. Nurses can provide more culturally sensitive care and improve patient satisfaction by acknowledging and accommodating these cultural preferences.

In Qatar, the healthcare system serves a diverse population, including Qatari nationals (Arab Muslims) and many expatriates from various countries [22]. Therefore, nurses in Qatar must be prepared to provide culturally competent care to patients from different cultural and religious backgrounds. For instance, understanding the importance of modesty and privacy in the Arab culture can help nurses in Qatar to communicate effectively and provide care that respects their patients’ values. One example of CRT in Qatar is seen in the maternity care setting. Qatari women often prefer female healthcare providers and respecting this preference can help build trust and rapport between the nurse and the patient [23]. Furthermore, understanding the significance of family and social support in Qatari culture can lead nurses to involve family members in the care process, enhancing patient satisfaction and health outcomes.

Singapore, a multicultural and multiracial country, presents unique challenges and opportunities for nurses to engage in CRT [24]. The three main ethnic groups in Singapore are Chinese, Malay, and Indian, each with distinct cultural beliefs, values, and practices [25]. Nurses in Singapore must be aware of these cultural differences to provide appropriate care to their diverse patient population. For example, traditional Chinese beliefs about health and illness often emphasize the balance between yin and yang energies, affecting patients’ treatment preferences and understanding of illness. Nurses in Singapore who are knowledgeable about these beliefs can incorporate them into their care plans, providing culturally sensitive and evidence-based care. Similarly, nurses working with Malay patients should be aware of the importance of religion and spirituality in their patients’ lives. Islamic practices, such as daily prayers and dietary restrictions, should be considered when developing care plans for Malay patients. Nurses can build trust with their patients and provide more effective care by demonstrating cultural sensitivity and understanding.

It is worthy to be mentioned that CRT is certainly applicable in any country where there is diversity, whether that diversity is in terms of regional, ethnic, linguistic, or religious differences [26]. The need for CRT increases even more in countries or regions with multinational or multicultural populations. This necessity reaches beyond multinational workforces, such as a diverse team of nurses from various countries, and includes the importance of understanding and addressing culturally diverse patient populations. In the case of China, there are 56 officially recognized ethnic minority groups [5], each with its unique cultural practices, languages, and beliefs. Therefore, implementing CRT in nursing education and practice in China could potentially improve healthcare delivery to these diverse groups [27]. By acknowledging and incorporating these differences into patient care, nurses can provide more compassionate, effective, and personalized care, thereby enhancing patient satisfaction and health outcomes.

Implementing CRT in diverse nursing workforces has challenges and obstacles, primarily arising from the intricacies of cultural differences and the necessity for systemic backing. Nurses may lack sufficient cultural awareness, even with the noblest intentions, leading to stereotypes, misunderstandings, or misinterpretations [28]. Language barriers can complicate crucial patient-nurse interactions, and although language services exist in many healthcare institutions, they may not be instantly accessible, and subtle language nuances could be lost in translation [29]. Balancing cultural sensitivity with evidence-based care presents a dilemma, as some traditional health beliefs and practices might conflict with scientific healthcare recommendations. Systemic barriers to CRT implementation may manifest as a lack of institutional support, limited resources, or inadequate recognition of the importance of culturally competent care.

Moreover, nursing curriculums might overlook an extensive focus on CRT. Given the pre-existing nursing shortage and high workloads, time and resource constraints in the healthcare setting can make it challenging to understand each patient’s unique cultural context fully. Lastly, defining cultural competence can be problematic due to its complex and multi-dimensional nature, thereby complicating its integration into nursing education and practice. Despite these hurdles, it is critical to note that implementing CRT in nursing is not only essential but feasible. Overcoming these challenges might require continuous cultural competency training [30], systemic policy changes supporting culturally responsive care, provision of language services, and commitment to patient-centered care. Persistent dialogue about these challenges and potential solutions is vital to ensure that healthcare remains accessible, appropriate, and effective for all patients, regardless of their cultural background.

The future of CRT in nursing holds exciting potential. As global migration trends continue, CRT will become increasingly critical in nursing education and practice. The focus will likely shift towards comprehensive inclusion of CRT in nursing curriculums globally. Research exploring the effectiveness of various CRT methodologies and their impact on patient outcomes will gain traction. Digital technology can also play a pivotal role in disseminating CRT education, transcending geographical boundaries. Ultimately, a more culturally competent nursing workforce can drive a transformative change in global healthcare, fostering more inclusive, patient-centered, and effective care for diverse populations.

In conclusion, CRT is a crucial pedagogical approach for nurses working in diverse workforces in terms of regional, ethnic, linguistic, religious, or nationality differences. Nurses can provide more effective, compassionate, and patient-centered care by recognizing and embracing the cultural differences of patients in various countries, including the US, Canada, Australia, the UK, Qatar, and Singapore (Fig. 1). In addition, implementing CRT strategies, such as self-reflection, cultural competency training, active listening, collaboration, and resource utilization, can help nurses develop the skills and knowledge needed to deliver culturally responsive care and improve health outcomes for their diverse patient populations.

**Fig. 1:**
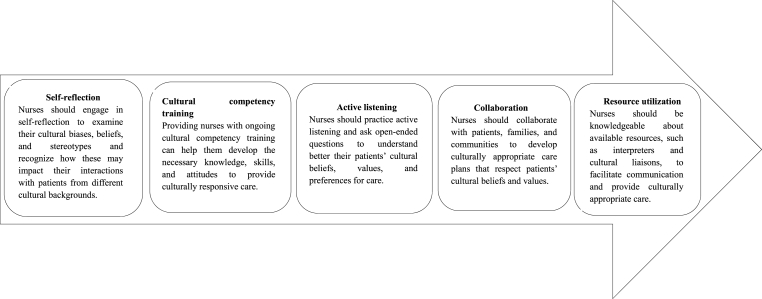
Suggested strategies to effectively implement the culturally responsive teaching in nursing practice.

## Declaration of competing interest

The authors have declared no conflict of interest.
